# Abiotic environmental conditions determine phage resistance outcomes in a salt-marsh bacterium

**DOI:** 10.1098/rstb.2024.0071

**Published:** 2025-09-04

**Authors:** Ella Silk, Kate Harding, Marina Mahler, Peter C. Fineran, Sean Meaden

**Affiliations:** ^1^ Department of Microbiology and Immunology, University of Otago, Dunedin 9016, New Zealand; ^2^ Department of Medicine, University of Otago, Christchurch 8140, New Zealand; ^3^ Bioprotection Aotearoa, University of Otago, Dunedin 9016, New Zealand; ^4^ Genetics Otago, University of Otago, Dunedin 9016, New Zealand; ^5^ Maurice Wilkins Centre for Molecular Biodiscovery, University of Otago, Dunedin 9016, New Zealand; ^6^ Department of Biology, University of York, York YO10 5DD, UK

**Keywords:** phage, resistance, abiotic environment, population sequencing

## Abstract

Phages exert strong selective pressure on bacterial hosts, yet the role of abiotic factors in resistance evolution is often overlooked. Abiotic effects can shape both demographic factors, such as encounter rates, and trade-offs between resistance and fitness. We used the salt-marsh bacterium *Prodigiosinella confusarubida* (a.k.a. *Serratia* sp. ATCC 39006) to examine how salt concentration shapes resistance evolution after exposure to a virulent myovirus phage (LC53), which infects via an outer membrane protein (OmpW). We find that resistance only emerges under low-salt conditions via mutations in the regulatory region upstream of *ompW* or within *ompW*. This effect was independent of phage type or receptor utilization, as similar resistance patterns appeared when using a flagella-tropic siphovirus (JS26). These findings suggest that indirect effects influenced by salt, such as bacterial and phage population sizes and encounter rates, play a crucial role. Overall, these results may help explain patterns of bacterial and phage genomic diversity in natural microbial communities, with consequences for predicting phage resistance evolution in applied contexts, such as phage therapy and the food industry.

This article is part of the discussion meeting issue ‘The ecology and evolution of bacterial immune systems’.

## 1. Introduction

The viruses that infect bacteria, bacteriophages, are ubiquitous in bacterial communities. It is, therefore, expected that they exert strong selective pressures on hosts to evolve resistance, either through the modification of surface receptors or via the acquisition of intracellular immune mechanisms [1–4]. In natural ecosystems, this interaction occurs in the presence of complex biotic and abiotic variables and likely shapes the evolutionary outcomes. In the case of surface receptor mutation, these surface receptors likely encode many functions that are critical for fitness in the environment (reviewed in [5]). It is, therefore, often assumed that while mutation, or loss, of a receptor may be beneficial for phage resistance, there will be a trade-off with fitness in the environment (although ‘trade-ups’ may also occur [6]). The notion of trade-offs is appealing for the clinical use of phages as it suggests that the evolution of resistance may lead to the subsequent loss of virulence or, in some cases, re-sensitization to antibiotics. For example, the evolution of phage resistance led to the re-sensitization of *Klebsiella* to beta-lactamase antibiotics [7], and phage resistance evolution has been linked to reductions in human, animal and plant pathogen virulence [8–11].

Receptor mutation, and subsequent evolution of phage tail fibres, appears to be a common, repeatable outcome of laboratory-based phage–bacteria coevolution studies [12]. However, it remains unclear how representative these observations are in natural environments and to what extent they are shaped by specific biotic and abiotic factors. For example, a study of the evolution of phage resistance within a plant system found that, while resistance readily arose *in vitro*, resistance evolution in the plant was negligible [13]. Moreover, mutations that conferred a substantial benefit *in vitro* did not realize the same fitness benefit in the plant [14]. The evolution of phage resistance in plantlets, via lipopolysaccharide (LPS) mutation, can also arise rapidly without inhibiting the virulence of a plant pathogen [15]. In addition, the associated cost of resistance with a surface receptor mutation in *Pseudomonas aeruginosa* was amplified when competitive biotic interactions were introduced, relative to a laboratory monoculture [16], suggesting that community context can be critically important for shaping resistance evolution [17]. Genomic data from environmental isolates suggest that phages may target receptors that are under strong purifying selection, meaning that any mutations are likely to be costly [18]. Further genomic analysis, coupled with infection assays, has identified surface receptors as the major determinant of phage host range, relative to intracellular defence systems [19]. Taken together, there are a range of fitness outcomes that are highly dependent on the environmental context.

Integrating environmental complexity is, therefore, important to predict outcomes in natural environments. For example, one study added bile salts to media to mimic the human gut environment, causing reduced encounter rates between bacteria and phages, which in turn weakened coevolution [20]. Our focal organism, *Prodigiosinella confusarubida* (a.k.a. *Serratia* sp. ATCC 39006), was isolated from a salt-marsh environment (Cheesequake, NJ, USA) and carries many traits suited to colonization of this environment, including the production of gas vesicles to allow migration to the air–liquid interface during low oxygen conditions [21] and flagellum-based swimming and swarming abilities [22]. Salt concentrations in similar salt-marsh environments vary between approximately 1 and 4% [23], which we hypothesized may shape the interaction between this bacterium and its phages. Here, we assess the role of salt in shaping the evolution of resistance to phages infecting a salt-marsh bacterium. Ultimately, understanding how abiotic conditions shape evolution is crucial for both natural microbial communities and phage therapy applications [24].

## 2. Methods

### (a) Strains and culture conditions

*Serratia* sp. ATCC 39006 LacA [25], referred to as WT herein, was used in this study along with derivative transposon mutants *ompW*::*Tn-DS1028uidAKm* (PCF613 and PCF615). The phage LC53 is a T4-like phage that uses the outer membrane protein OmpW as a receptor, has an arabinosylated genome [26] and was isolated from wastewater in Dunedin, New Zealand prior to this study (see [27] for isolation details). The phage JS26 is a flagella-tropic siphovirus also isolated from the same location [28]. *Serratia* sp. ATCC 39006 strains were cultured in lysogeny broth (LB, 10 g l^−1^ tryptone, 5 g l^−1^ yeast extract and either 5 g l^−1^ or 40 g l^−1^ NaCl for the 0.5 and 4% NaCl media respectively) unless otherwise stated and incubated at 30°C. LC53 and JS26 phages were propagated by selecting a single plaque following spotting onto LB agar (LBA) overlays seeded with *Serratia* (100 μl) using the tube-free agar overlay protocol described in [29]. Hard agar was made using 1.5% w/v bacteriological agar, and soft (top) agar used 0.35% w/v bacteriological agar.

### (b) Serial passage evolution experiments

Glass universals were prepared with either 0.5 or 4% NaCl LB medium to a volume of 5 ml. A serial passage experiment was initiated with 5 μl WT overnight culture (approx. 4.5 × 10^8^ colony-forming units (CFU) ml^−1^ ) and either 10 μl of phage stock (approx. 4.5 × 10^2^ plaque-forming units (PFU), multiplicity of infection (MOI) 0.0002) or 10 μl LB as a control. To eliminate pre-existing variation, replicate populations were initiated from a single clonal ancestor. This fully factorial design gave six replicates of each phage treatment, salt-level combination (*n* = 24 total), which were passaged daily using 1 : 100 dilutions after overnight incubation in a 30°C shaken incubator. About 10 μl of phage stock or 10 μl LB was added to the relevant treatment groups after each transfer. An additional 0.5% NaCl LB universal containing no bacteria or phage was passaged to detect contamination. Transfers were done every 24 h for 7 days using sterile filter pipette tips. Optical densities (600 nm) were recorded with 200 μl of each sample daily using the Varioskan Flash plate reader (ThermoFisher, USA). These plates were then used to measure phage titres following centrifugation (4°C, 3000*g*, 10 min) to pellet bacterial cells and 10-fold serial dilution of phages. About 5 μl was spotted onto lawns of *Serratia* as described above. Samples were frozen on days 0, 2, 4, 6 and 7 in 20% v/v glycerol and stored at –80°C until phenotyping and DNA extraction. The same experimental design was repeated for a subsequent experiment using the phage JS26.

### (c) Phage resistance streak assays *in vitro*

Bacterial culture samples were thawed and plated following serial dilution. After 48 h incubation at 30°C, 20 colonies were picked at random and inoculated into 200 μl LB in 96-well plates, then incubated overnight. Thirty microlitres of high-titre phages stock (3.3 × 10^10^ PFU ml^−1^ ) were spread across a square LBA plate in a vertical line. Each culture was then streaked across these lines at 90° using a multichannel pipette dipped into corresponding wells (24 colonies per square plate). Plates were incubated at 30°C for 24 h and were scored as follows: sensitive (reduced or no bacterial growth over the phage streak) or resistant (no reduction in bacterial growth over the phage streak).

### (d) Swimming assays

Tryptic swim agar (TSA, 10 g l^−1^ tryptone, 5 g l^−1^ NaCl, 3 g l^−1^ agar) [1] was used for swimming assays. Colonies from day 4 samples were tested for swimming ability to test flagellum viability in bacterial populations after the evolution experiment. Square plates were marked into six sections and 20 ml TSA was poured into each plate. Plates were allowed to set on the bench for 20 min to allow setting without over-drying. Three colonies from each replicate plate were picked using sterile toothpicks and stabbed into a sectioned area of the agar. Plates were incubated, lid up, for 20 h at 30°C. Swimming was measured by visual assessment as indicated by visible colony expansion in a circle, with surfactant production, and scored as swimming or non-swimming (see electronic supplementary material, figure S1).

### (e) Genomic DNA extraction and sequencing

DNA was extracted from the entire community on day 4 of the evolution experiment, by which time bacterial densities had recovered, suggesting resistance evolution. The ancestral population used to seed the starting cultures was also extracted as a control. Extraction followed the protocol of the DNeasy kit (Qiagen), using the Gram-negative bacteria extraction protocol, with minor adjustments: addition of 20 μl of proteinase K for maximum cell lysis, and 30 μl of prewarmed (50°C) AE elution buffer in place of 200 μl after an additional drying step (17 000*g* for 1 min) after the AW2 wash step. Sample purity and concentration were determined by Nanodrop (ThermoFisher) and the Qubit broad range dsDNA kit (ThermoFisher) and samples were sequenced using the MiGS service (Pittsburgh, PA, USA). From one of the populations that acquired the *ompW* promoter single-nucleotide polymorphism (SNP) at high frequencies (approx. 70%), based on the population-level whole-genome sequencing (population-seq) data, 10 clones were picked and the promoter region amplified by polymerase chain reaction (PCR) and Sanger sequenced (F: 5′ TTTTCTAGACCTAAATGAATCCTGTGAC 3′, R: 5′ GATGGAGTTCTGAGG- TCATTACTGGGCCGTACAATAACTAAGCGA 3′).

### (f) NaCl growth assays

Growth in 4 and 0.5% w/v NaCl LB was tracked as 24 h growth curves in 96-well plates, by means of a spectrophotometer (CLARIOstar, BMG Labtech) measuring OD_600_ absorbance. The WT and *ompW* mutants were assayed with either the *ompW* complement or an empty vector control using 0.1 mM isopropyl β-d-1-thiogalactopyranoside (IPTG) to induce expression. The WT was also compared with an *flhD* mutant and a clone carrying the *ompW* promoter SNP identified from the population-seq data under 4 and 0.5% NaCl conditions. For all growth curves, overnight cultures were standardized to 0.1 OD_600_, with measurements taken every 12 min for a 24 h period. Three biological replicates were included for each treatment. Two *ompW* transposon mutants (PCF613 and PCF615) were compared with the WT under a range of NaCl concentrations: 0.5, 3, 4, 5 and 6%. The functional role of *OmpW* was assessed using a Gen III microplate (Biolog, CA, USA). Each well has a unique biochemical environment including antibiotic resistance and various carbon sources. Cultures of *Serratia* sp. ATTC 39006 WT and the *ompW* transposon mutants were plated and grown at 30°C for 2 days. Two colonies were suspended in 12 ml of Biolog Inoculating Fluid to give an absorbance of 0.006 at 590 nm. One hundred microlitres of inoculant was added to each well and incubated at 30°C overnight. During incubation, respiration and growth are indicated by the formation of a purple coloration as increased respiration causes the reduction of a tetrazolium redox dye. Absorbance was measured at 750 nm, and values were compared between the strains.

### (g) Data analysis

Sequence data were quality trimmed using Sickle [30]. The *breseq* pipeline [31] was used for mapping and SNP calling, using the *Serratia* sp. ATCC 39006 LacA reference genome (Genbank accession: GCA_002847015.1). To detect variation within our populations, *breseq* was run using the ‘polymorphism’ mode, which identifies mutations present at frequencies greater than 5%. Only mutations present at frequencies ≥30% were used for downstream analysis. For phage genome analysis, a minimum coverage of 20 was required for mutation identification. Data analysis was performed using RStudio v. 4.1.2 (http://www.posit.co). R packages used were ‘tidyverse’ [32], ‘readxl’ [33], ‘stringi’ [34] and ‘htmltab’ (https://github.com/crubba/htmltab).

## 3. Results

### (a) Phage resistance evolution is inhibited by high NaCl concentrations

**Figure 1.**
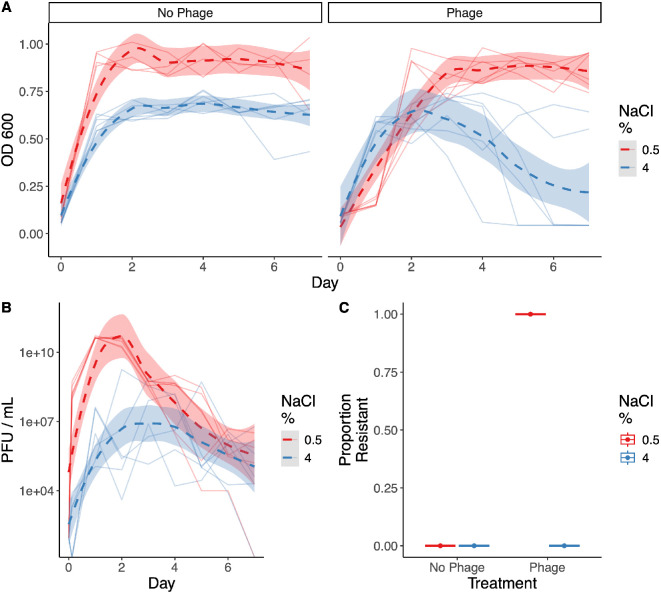
High NaCl concentrations reduce bacterial growth, phage LC53 titres and resistance evolution. (A) Optical densities (OD_600_) on each day of an *in vitro* evolution experiment for the no-phage treatment (left) and phage treatment (right). Colour denotes NaCl concentration used in the medium. Individual solid lines represent distinct populations, dashed line represents ‘loess’ model fit and shaded areas represent 95% confidence intervals. (B) Phage titres throughout the evolution experiment for the phage treatment lines. Individual lines represent counts from each population, dashed lines represent loess model fits and shaded areas represent 95% confidence intervals. No phages were detected in the phage-free selection lines. (C) Resistance evolution in each population from 24 colonies per population.

We aimed to assess whether high-salt concentrations, comparable to salt-marsh environments, affect the evolution of phage resistance. We ran a 7 day evolution experiment that repeatedly exposed an initially sensitive population of bacteria to the phage LC53 in high- (4%) or low-salt (0.5%) conditions and tracked the bacterial and phage dynamics (figure 1A,B). We screened for resistance at day 4 and found widespread resistance had evolved. However, resistance was confined to the low-salt, phage-exposed experimental lines only. All colonies from the low-salt, phage-exposed lines showed resistance to the ancestral phage by day 4 (figure 1C; 24 colonies per population). By contrast, no colonies from the high-salt, phage-exposed lines showed resistance by this time (figure 1C), despite high phage titres throughout the experiment (figure 1B). This result supports the hypothesis that the abiotic environment shapes the frequency at which phage resistance evolves.

### (b) Mutations linked to outer membrane protein arise in phage resistant populations

**Figure 2.**
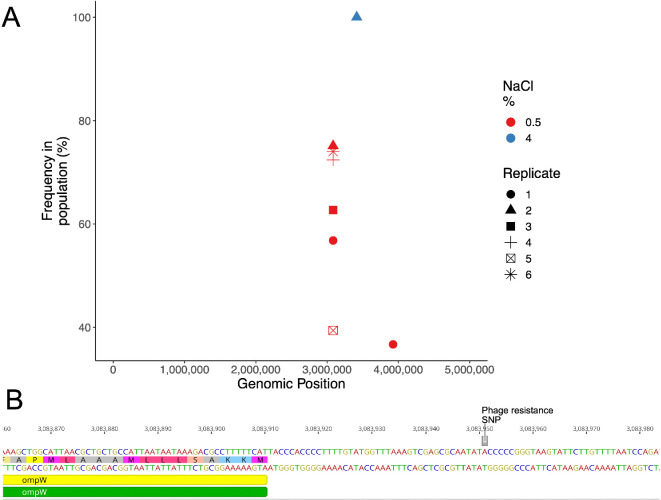
Promoter mutations arise in the region upstream of *ompW*. (A) Population-level whole-genome sequencing identified mutations in the intragenic region upstream of *ompW* present in >30% of the population. Shapes denote independent replicate populations and colour denotes NaCl concentration used. Average coverage for each replicate was approximately 100×. (B) Highest-frequency single-nucleotide polymorphism (SNP) (A→C) located 41 nt upstream of *ompW*.

To identify the mutations responsible for resistance, we performed population-level whole-genome sequencing (population-seq). By sequencing at the population level, we could identify mutations present at high frequencies (>30%) with high confidence. Lower-frequency mutations (>5%) are available in electronic supplementary material, table S1. In all populations that evolved phage resistance, there were mutations in the promoter region of the *ompW* gene (table 1, figure 2A); there were no mutations in (or near) this gene in the populations that did not evolve phage resistance (figure 2A). Interestingly, an identical mutation reached high frequencies in all low-salt, phage-exposed populations. This mutation is at position 3 083 951 in the promoter region, 41 bases directly upstream of *ompW* (figure 2B). Sanger sequencing of *ompW* and the region directly upstream validated the SNP frequencies identified by population-seq from 10 randomly selected colonies within a selected experimental population: 6/10 had the identified promoter region SNP, 2/10 had a mutation in the *ompW* gene and 2/10 were WT. An efficiency of plaquing assay showed this promoter mutation conferred complete resistance, equivalent to an *ompW* transposon mutant (electronic supplementary material, figure S2). The presence of an identical mutation in all six replicate populations suggests that the mutation was likely present at low frequency in the founding population used to seed the experiment; however, it did not reach a detectable frequency in the control lines, and the presence of additional *ompW* mutations in the same lines suggests strong selection for this particular mutation under these conditions when phage titres are high (electronic supplementary material, table S1).

**Table 1.** All mutations at day 4 with >30% frequency from population sequencing of evolutionary lines. Table includes mutation details and the associated replicate population.

genome position (base)	mutation	frequency (%)	annotation^a^	description	replicate^b^
3083951	A→C	75.1	intergenic	outer membrane protein	P_0_2
3083951	A→C	74	intergenic	outer membrane protein	P_0_6
3083951	A→C	72.4	intergenic	outer membrane protein	P_0_4
3083951	A→C	62.7	intergenic	outer membrane protein	P_0_3
3083951	A→C	56.8	intergenic	outer membrane protein	P_0_1
3083951	A→C	39.4	intergenic	outer membrane protein	P_0_5
3415436	A→C	100	intergenic	flagellar-brake protein	P_4_2
3925586	C→G	36.7	intergenic	NADP-dependent phosphogluconate dehydrogenase	P_0_1

^a^ Type of mutation identified.

^b^ P, coevolved with phage; 0/4, Nacl% concentration; numbers 1–6, evolutionary line.

We also mapped the population-seq data to the LC53 phage genome. Zero reads mapped to the phage genome in the ‘no-phage’ treatment, confirming there was no contamination, and there was no significant difference in the overall phage mutation counts (electronic supplementary material, figure S3). We identified two common phage mutations in the phage-exposed populations, namely in a tail fibre adhesin and holin protein-encoding gene. Tail fibre adhesion is a critical step in infection, and holins are proteins linked to the timing of phage lysis from the cell; therefore, these mutations are consistent with selection for infectivity traits. However, as they occurred in almost all phage-exposed populations, we cannot exclude the possibility that they arose during the outgrowth phase of preparation of phage stocks prior to seeding the experiment. Together these results show that resistance to LC53 is achieved by mutation of *ompW*, most commonly in the promoter region, but other mutational routes to resistance are likely, based on the additional *ompW* SNPs, and these mutations lead to a reduction in phage titres after an initial period of successful infections.

### (c) NaCl inhibits bacterial growth and outer membrane protein does not provides substantial salt tolerance in *Serratia* sp. ATCC 39006

**Figure 3.**
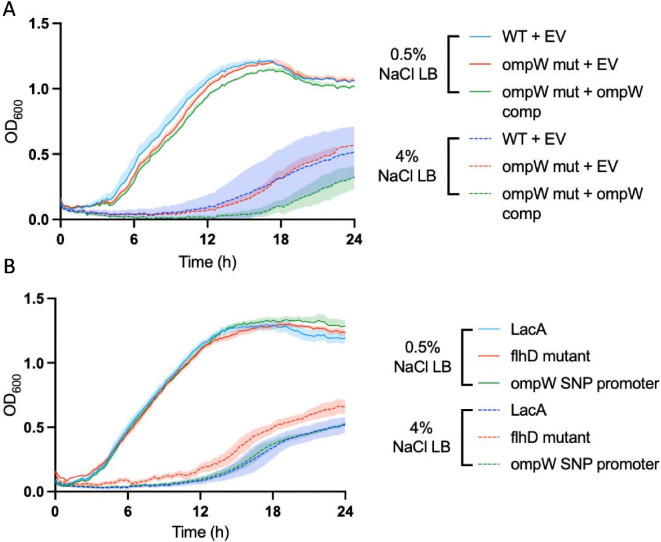
OmpW does not confer a benefit under high-salt conditions. (A) Growth rate assays of wild-type (WT) and an *ompW* mutant with an *ompW* complement or an empty vector (EV) control at 4 and 0.5% NaCl. (B) Growth rate assays of the WT LacA strain, the *ompW* promoter SNP mutant and an *flhD* (flagella) mutant at 4 and 0.5% NaCl. Lines represent the mean of three biological replicates, with shaded areas showing ±s.d.

Given that we only observed *ompW* mutations in the low-salt lines, we hypothesized that OmpW may play a vital role in conferring salt tolerance, and therefore mutations are not viable, even in the presence of phages targeting this receptor. By comparing *Serratia* sp. ATCC 39006 with an isogenic *ompW* transposon-mutant derivative [27], we assessed the functional role of OmpW. We compared the growth of WT and two independent *ompW* transposon mutants across a range of NaCl concentrations (0.5–6%; electronic supplementary material, figure S4). While growth was inhibited with increasing NaCl concentrations, there were no appreciable differences between the strains, suggesting OmpW does not play a major role in salt tolerance in rich media (figure 3A). We next compared the *ompW* transposon mutants that had been complemented with *ompW* and again observed that high salt concentrations inhibited growth, but the *ompW* complement conferred no advantage over the empty vector control (figure 3B). To assess whether more subtle changes in *ompW* expression were facilitating salt tolerance, we compared a clone carrying the *ompW* promoter region SNP with the WT, but again found no difference between the strains and overall growth inhibition under high-salt concentrations. To identify any other possible functions of OmpW, we compared the WT with an *ompW* transposon mutant using a BIOLOG assay plate, which assesses 94 unique biochemical tests. With this assay, we observed almost no differences between the strains (electronic supplementary material, figure S5). Interestingly, we did find growth inhibition of the *ompW* transposon mutant in the 8% NaCl test but not the 4% NaCl test. However, our previous experiments found complete growth inhibition at NaCl concentrations of 6%. Therefore, while we cannot entirely rule out a role for *ompW* in salt tolerance, our experiments that most closely resemble the serial passage conditions find no evidence for salt tolerance affecting *ompW* evolution.

### (d) High NaCl conditions prevent the evolution of flagella-based phage resistance

Our assays suggested that a trade-off between salt tolerance and phage resistance, via the OmpW receptor, was not the cause of the differences we observed in mutational outcomes between high and low salt concentrations. Reduced phage stability under high-salt conditions could also explain our results, but we ruled this out by measuring phage titres for 48 h in the different conditions in the absence of bacteria. Although there was a general decline in titres over time, LC53 appeared to be more stable in the high-salt medium (electronic supplementary material, figure S6) than the low-salt medium, suggesting phage stability is not affecting our results. We observed that bacterial densities were lower in the high-salt environment in the no-phage control lines (figure 2A,B), meaning indirect effects, such as lower encounter rates between bacteria and phages or reduced mutation supply, could also play a role in shaping resistance. We therefore hypothesized that alternative factors, such as reduced encounter rates, might prevent the evolution of resistance. If so, this would lead to similar results if we repeated the experiment with another phage that targets an alternative receptor. To test this, we ran a subsequent experiment with a phage (JS26) that uses the flagellum as a receptor [28]. All else being equal, flagella mutations should be acquired equally in both high- and low-salt environments. Mutations should also carry minimal cost owing to the shaking, liquid environment used in this experimental design, where flagella are unlikely to be functionally beneficial. We first verified that any receptor effects were independent between experiments by confirming that the OmpW-targeting phage, LC53, did not use flagella as an alternative receptor. An efficiency of plaquing assay of LC53 infection was performed on the WT with a CRISPRi knockdown of *flhD* and an empty vector control (electronic supplementary material, figure S7; [35]) and found no differences, indicating flagella are not involved in successful LC53 infection. A growth assay subsequently confirmed similar growth inhibition of an *flhD* transposon mutant under high-salt conditions (figure 3B).

**Figure 4.**
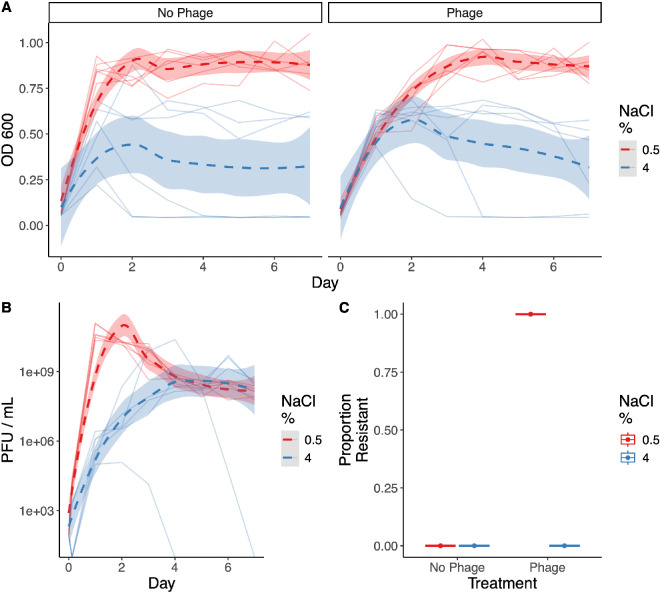
Impact of NaCl and JS26 phage treatments on population phenotypes. (A) Daily optical densities on each day of the evolution experiment for the no-phage treatment (left) and phage treatment (right). Colour denotes NaCl concentration used in the medium. Solid lines represent distinct populations, dashed line represents ‘loess’ model fit, and shaded areas represent 95% confidence intervals. (B) Phage titres throughout the evolution experiment for the phage treatment lines. Individual lines represent counts from each population, dashed lines represent ‘loess’ model fits and shaded areas represent 95% confidence intervals. No phages were detected in the phage-free selection lines. (C) Resistance evolution in each population from 24 colonies per population.

Similar to our experiments with LC53, resistance to JS26 evolved readily in the low-salt environment but not in the high-salt environment (figure 4). Further, the overall bacterial and phage growth dynamics were largely comparable with the previous experiment with LC53 (figure 1). To assess whether resistance was due to a loss of flagella function, we conducted swimming assays in soft agar and scored the results visually (electronic supplementary material, figure S1). We found all colonies isolated on day 4 of the passage experiment from the 0.5% NaCl, phage-exposed lines showed reduced swimming activity (*n* = 18). In contrast, no colonies isolated from the non-resistant populations (4% NaCl lines and 0.5% NaCl, no-phage lines) showed any inhibition in their swimming activity (*n* = 39). We therefore conclude that phage resistance to JS26 in these lines is conferred by the functional loss of flagella. Taken together, these results support the hypothesis that resistance evolves as a result of indirect effects arising from the abiotic environment, such as reduced encounter rates between bacteria and phage, rather than direct trade-offs between growth and resistance linked to the phage receptor.

## 4. Discussion

Previous studies have identified trade-offs between competitive fitness and phage resistance (reviewed in [6]). Yet the role of abiotic conditions that bacteria experience in natural environments in shaping resistance has been relatively understudied. By performing serial passage experiments using environmentally relevant salt concentrations, we assessed how frequently phage resistance arose in a salt-marsh bacterium. In our evolution experiments to assess the mutational outcome of exposure to a lytic phage across high- and low-salt concentrations, resistance only evolved under low-salt conditions. These mutants arose most commonly via mutations in the promoter region of *ompW*, but also within the gene at a lower frequency. Although phage resistance was observed in all screened colonies under low salt, we observed variation in the frequencies of the promoter mutation, and this mutation did not reach fixation in any populations. This lack of mutation fixation suggests that either additional mutations are present at lower frequencies, undetectable by whole-population sequencing, or subpopulations of susceptible cells can coexist with a resistant population, as has been observed in long-term evolution experiments with phage lambda [36], and are indicative of herd immunity [37]. Both modelling and experiments found a threshold of 75% of phage-resistant cells is required to prevent extinction, but this threshold reduces if bacterial growth is slower [38], which is a plausible scenario under our experimental conditions. Importantly, herd immunity in non-structured environments requires resistant bacteria to act as a sink for infections [38]. Our finding of mutation in the promoter region suggests changes in *ompW* regulation and expression rather than complete receptor loss. Previous work found that the Rcs stress pathway reduces *ompW* expression alongside adaptive immunity and provides resistance to LC53 [39]. Understanding the triggers of this pathway may have consequences for when *ompW* mutations are most likely to arise and implications for population-level protection.

Similar results with the flagella-binding phage JS26 demonstrate that *ompW* mutations did not arise as a result of a trade-off between growth and resistance. Instead, indirect effects, such as contact rates, are likely preventing resistance evolution, as shown during evolution of phage resistance in *Escherichia coli* under the addition of bile salts [40]. While trade-offs may occur in many scenarios, our results suggest that indirect effects, resulting from population dynamics, can play a substantial role in determining when resistance arises. We also see equivalent growth between phage and no-phage controls under high-salt conditions in some of the selection lines (figures 1A and 4A), suggesting insufficient phage infections to select for resistance. Similarly, previous work in a marine *Vibrio* and filamentous phage system has found suboptimal salinity levels constrain resistance evolution owing to reduced growth rates [41]. Mathematical modelling has also described replication thresholds, whereby a certain density of susceptible hosts is required for a phage epidemic to occur [42]. We suggest these considerations be taken into account for predicting the evolution of resistance in phage therapy applications.

Although our high-salt condition is environmentally realistic, this value is at the upper bounds of salt-marsh measurements [23]. Both seasonal and spatial variation may lead to heterogeneity in the salt concentrations experienced by bacteria in these environments. Environmental variation may change the selective forces that shape bacteria and phage coevolution, potentially leading to the coexistence of diverse bacterial populations that vary in their receptor allelic diversity. However, further work is required to test more fine-grained differences in salt concentration and variation in natural environments. In the more homogeneous marine environment, genomic data suggest that phages target receptors that are under strong purifying selection [18]. Assessing whether this is also the case across heterogeneous environments will be valuable future work, although *ompW* appears to be highly conserved across many bacterial families, including Enterobacteriaceae and Vibrionaceae, with numerous putative functions [43]. LC53 is also capable of infecting various pectobacteria, demonstrating cross-genera infection and that OmpW targeting is a widespread infection strategy [27].

We note that, in our experiment, we repeatedly added an ancestral phage stock, removing the ability to detect coevolutionary dynamics. This is an important point as it remains to be seen how the abiotic environment may limit the ability of the phage to counter-adapt to the loss or modification of the OmpW receptor. If populations are smaller, then adaptation (and counter-adaptation) will likely be slower, owing to reduced mutational supply [44]. Ultimately, differentiating abiotic and biotic effects is challenging as these components are usually linked [20]. We did not see resistance arise later in the high-salt condition, suggesting the lack of resistance evolution is due to more than mutation supply. However, further work to understand whether resistance may arise later (beyond 7 days) under high-salt conditions may advance our understanding of how bacterial and phage populations coevolve.

The results presented here demonstrate that abiotic factors limiting bacterial growth can determine evolutionary outcomes for phage resistance. These results are also of consequence to the use of phages in the clinic or food industry and aid predictions of the underlying factors that shape the evolution of resistance. It is already established that biotic factors such as phage density can determine the relative benefits of surface-based resistance over intracellular resistance [45]. Integrating knowledge of how abiotic factors shape this interaction may allow better predictions of how, and when, resistance will evolve in applied settings, and ultimately allow minimizing resistance evolution or directing resistance towards therapeutically beneficial outcomes [24,46], such as re-sensitization to antibiotics [7].

## Data Availability

Assay data are available from the supplementary file ‘nacl_experimental_evolution_data.xlsx’. Raw sequence data are available from the European Nucleotide Archive (ENA) under accession PRJEB51326. Code for analysis is available at https://github.com/s-meaden/Silk_abiotic_evo and a Zenodo repository [47]. Supplementary material is available online [48].
